# Epigenetic reprogramming of hepatic antigen presenting cells in chronic liver disease

**DOI:** 10.3389/fimmu.2026.1783588

**Published:** 2026-04-20

**Authors:** Enrique Ángel-Gomis, Oriol Juanola, Esther Caparrós, Rubén Francés

**Affiliations:** 1 Hepatic and Intestinal Immunobiology Group, Dpto. Medicina Clínica and Instituto Instituto de Investigación, Desarrollo e Innovación en Biotecnología sanitaria de Elche (IDIBE), Universidad Miguel Hernández, San Juan de Alicante, Spain; 2 Instituto de Investigación Sanitaria y Biomédica de Alicante (IIS ISABIAL), Hospital General Universitario Dr. Balmis, Alicante, Spain; 3 Centro de Investigación Biomédica en Red. Enfermedades Hepáticas y Digestivas (CIBERehd), Instituto de Salud Carlos III, Madrid, Spain

**Keywords:** chronic liver disease, epigenetic modifications, fibrosis, Kupffer cells, LSECs

## Abstract

Chronic liver disease (CLD) is a high morbidity and mortality condition with a high incidence/prevalence worldwide. Despite the diverse etiological factors involved, common pathogenic hallmarks in CLD progression are the accumulation of scarring hepatic tissue and a sustained local and systemic inflammatory response. Considering the constant exposure of the liver to antigens and the delicate homeostatic immune surveillance that takes place in the organ, hepatic antigen presenting cells (hAPCs; i.e. Kupffer Cells, Liver Sinusoidal Endothelial Cells and Dendritic Cells) and their activity regulation are key players in the maintenance of intrahepatic immune tolerance. Epigenetic regulation is considered as a relevant mechanism in several stages of CLD development, currently recognized for playing a pivotal role in hepatic stellate cells activation in liver fibrosis as well as tumor suppressor gene silencing and tumoral microenvironment immune-escape. However, influence of epigenetic mechanisms over immune response in CLD continues under study. Here we comprehensively review the main epigenetic regulatory mechanisms controlling hAPCs regulation, considering the intricate crosstalk of epigenetic effectors and discussing recent studies supporting epigenetic interventions with promising therapeutical potential over inflammatory response during CLD progression.

## 1 Regulation of hepatic immune response in chronic liver disease

The liver is the largest solid organ in human body and is responsible for carrying out a myriad of biological functions, highlighting protein and lipid metabolism, detoxification, nutrient storage and protein synthesis. It is also well known that the liver is home of a complex immunological activity, including a strong innate immune capacity, poor adaptive immune response versus over-reactive autoimmunity and, specially, induction of local and systemic immune homeostasis (1, 2).

Hepatic immune tolerance maintenance is exerted by means of differentiating damaging and harmless blood-borne antigens at sinusoidal and perisinusoidal spaces (i.e. the space of Disse). In this anatomical location, resident lymphocytes and hepatic antigen presenting cells (hAPCs) maximize pathogen and gut-derived antigen screening. hAPCs include cells from the reticulo-endothelial system of the liver: Kupffer Cells (KCs) and Liver sinusoidal Endothelial Cells (LSECs); and Hepatic Stellate Cells (HSCs), located in the perisinusoidal area delimited between hepatocytes and LSECs; as well as liver Dendritic Cells (DCs) (3, 4). hAPCs interact with a wide variety of hepatic immune cell populations, highlighting Natural Killer T cells, innate lymphoid cells and γδ T cells.

Chronic Liver Disease (CLD) is a high morbidity and mortality condition with considerable prevalence in several countries, regardless their socio-economic environment. Multitude of maintained insults such as alcohol and drug abuse, metabolic-associated liver disease and viral hepatitis can lead to a progressive loss of organ function, that initially shows few or no symptoms (the so called “compensated” stage of CLD) but ultimately evolves to the extensive liver damage and life-threatening complications that characterize Advanced Chronic Liver Disease (ACLD, also known as “decompensated” stage of CLD) (5, 6). From an anatomical perspective, during CLD progression, the excessive accumulation of extracellular matrix, mainly deposited by activated HSCs (7), facilitates the replacement of functional, healthy liver tissue architecture by regenerative hepatic nodules.

Tolerogenic immune liver function is compromised and eventually impaired during CLD progression. Sinusoidal and septal fibrosis, LSECs capillarization and KCs reduction in number and functional capacity reduce their antigen presenting functions, necessary for a proper liver immune surveillance (3). As for DCs, these highly specialized antigen-presenting cells play crucial role in fibrosis progression by regulating HSCs myofibroblastic differentiation as well as affecting liver immune response by interplaying with several cell populations (8). The secretion of damage-associated molecular patterns by necrotic hepatocytes and proinflammatory cytokines by HAPCs and recruited lymphocytes contribute to the permanent activation of HSCs and causes a constant activation of the immune system (9). The continuous bacterial and antigen challenge characteristic of homeostatic liver plus the persistent inflammatory state of CLD are responsible of a dissociation between the regulatory and effector immune response (1), thus generating both a systemic increase in exposure to bacterial antigens and gut microbes and a final exhaustion of immune systems response capacity due to persistent hyperactivation. The paradoxical situation of systemic inflammation and local immunodeficiency is mediated by a complex network of alterations comprising intracellular signaling pathways and metabolism, intercellular interactions and epigenetic changes (10).

As in any other tissue, normal liver activity relies on the functional properties of both its constituent cells as well as non-parenchymal cells, where maintenance of phenotypic traits is tightly associated to the proper function development. Epigenetic mechanisms have acquired a relevant role in CLD progression, recognized as a central figure in HSCs phenotypic changes during their activation (11), and contribution to liver fibrosis by extracellular matrix deposition (12). Epigenetic dysregulation of tumor suppressor genes expression has also been described in hepatoblastoma, enabling the establishment of epigenetic variants with clinical implications (13–15). However, most of these studies focus their research on hepatocytes, the most abundant cell type in the liver and the responsible of the organ physiological main function; and hepatic stellate cells, due to their role as primary drivers of liver fibrosis after their transition into an activated, proliferative, collagen-producing myofibroblastic type upon chronic liver injury. This situation leaves a knowledge gap regarding the epigenetic regulation of several hepatic populations, namely APCs like KCs, LSECs and DCs. In these circumstances, we wanted to specifically focus our review on the aforementioned cell-types with the aim of enlightening their epigenetic profiling during disease progression, which remains poorly understood.

## 2 Epigenetic mechanisms for regulation of gene expression

### 2.1 General considerations

**Figure 1:**
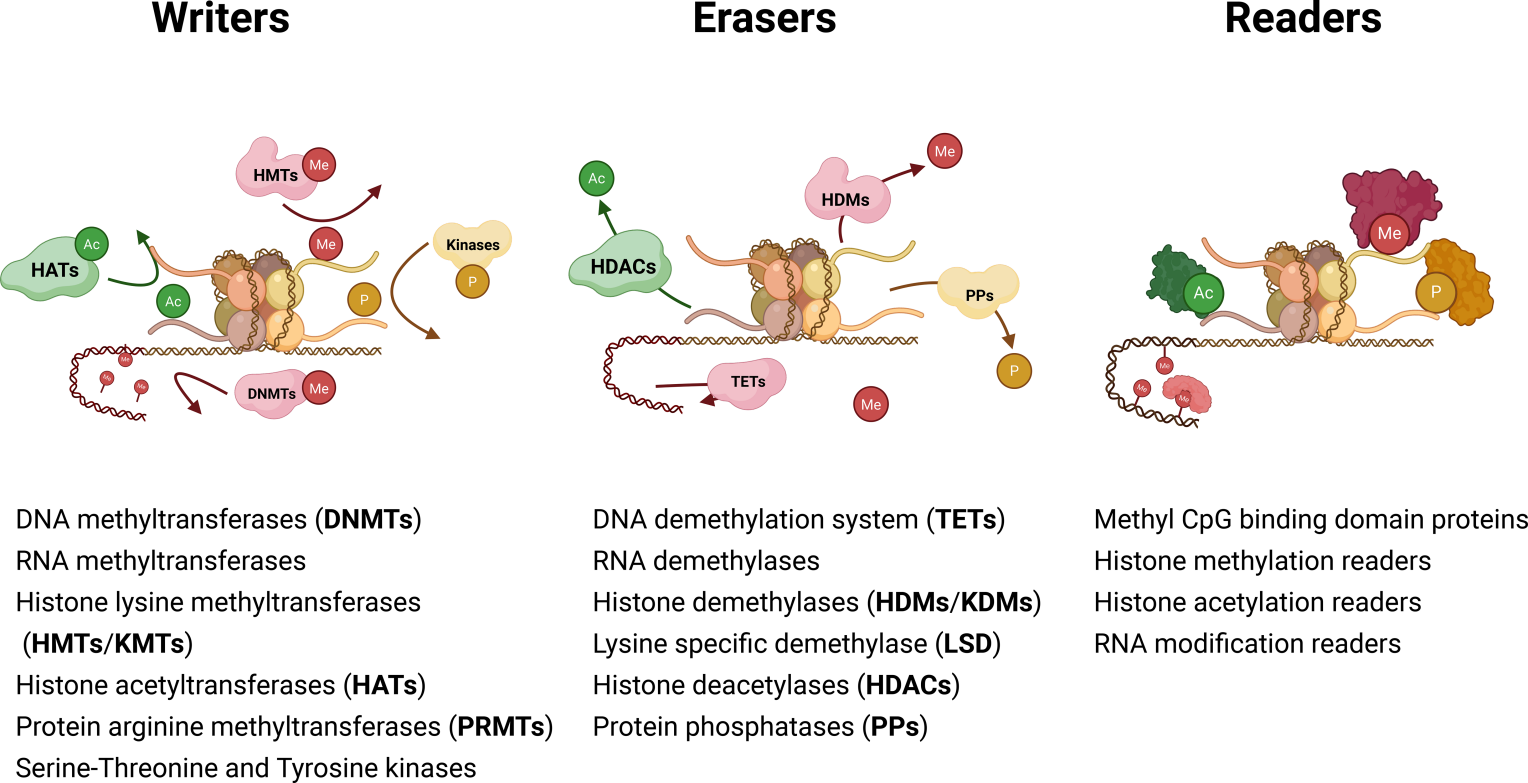
Primary epigenetic mechanisms regulating Open/Closed chromatin state transitions and gene expression. Diagram shows the different levels of genetic material organization and processing, ranging from single nucleotides to DNA transcription and mRNA translation up to chromatin nucleosomal ultrastructure and nuclear proteins association. Major epigenetic regulatory mechanisms are depicted, diagrammatically indicating their action range with respect to aforementioned genetic material organization levels. Figure has been modified from Fernández-Barrena MG et al. *Semin Immunol 2025: 101980* and created with Biorender (https://www.biorender.com/).

**Figure 2:**
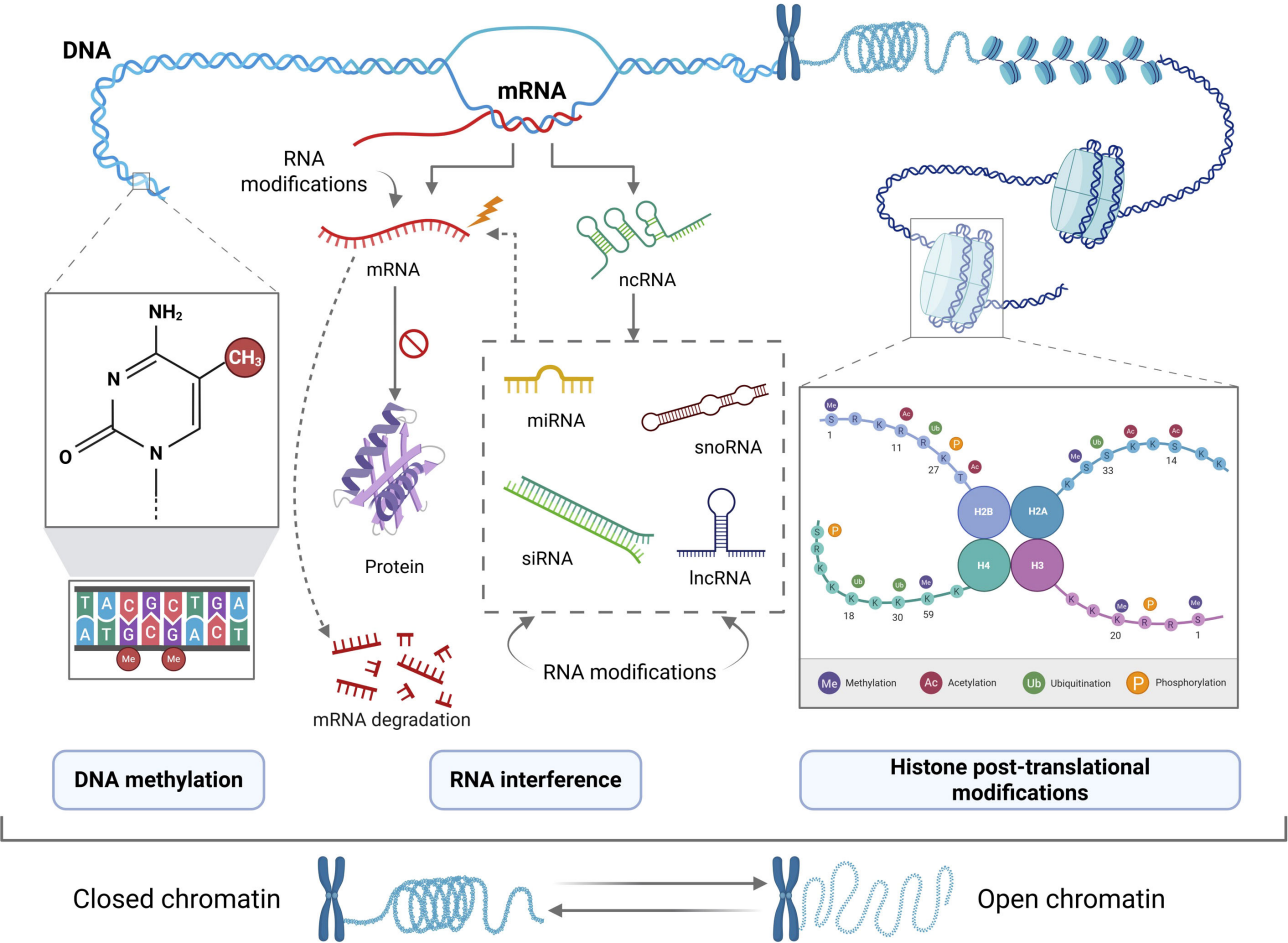
Main epigenetic effectors: writers, erasers and readers, involved in chromatin conformation and gene expression regulation. Schematic representation of principal epigenetic effectors that regulate hepatic antigen presenting cells during CLD progression, classified according their role in depositing (Writers), eliminating (erasers) or binding (readers) epigenetic marks. Figure has been created with Biorender (https://www.biorender.com/).

Epigenetic reversible mechanisms regulate changes in the ultimate manifestation of a locus or chromosome without altering their underlying sequence. The epigenetic regulation of gene expression is an utterly dynamic process mainly controlled by three enzyme groups that act as key players. *Writers* are responsible for epigenetic mark deposition; conversely, *erasers* oversee the elimination of those marks. However, the outcome of gene expression or silencing due to the presence (or absence) of a determined set of epigenetic marks eventually depends on *readers*, a diverse range of proteins that are responsible of the recognition of epigenetic marks in a locus (16). Epigenetic regulation of gene expression comprises several mechanisms which are typically grouped as DNA methylation, Histone modifications and non-coding RNAs (ncRNAs) (**Figure 1**). Epigenetic regulators mentioned in this work are summarized in **Figure 2**.

### 2.2 DNA methylation

One of the most extensively studied chromatin modification is cytosine methylation. This process takes place with the covalent binding of a methyl group at the cytosine’s 5^th^ carbon within a cytosine-phospho-guanine (CpG) dinucleotide, usually forming dinucleotide clusters in the so called CpG islands (17, 18). Although classically associated to gene silencing, CpG methylation is known to have different effects in gene regulation depending on location of methylated CpG and their basal state. As a rule, hypermethylation of CpGs located in gene promoters or first exon result in gene repression. In contrast, it has been described that hypermethylation of lowly methylated CpG islands or gene bodies shows gene activation as final outcome (19). This dynamic epigenetic modification is carried out by two groups of enzymes with antagonistic activities. On one hand, DNA methyltransferases (DNMTs) are enzymes responsible for either *de novo* methylation (DNMT3A and DNMT3B) or methylation pattern maintenance after cell replication (DNMT1) (20). Nevertheless, there is accumulative evidence pointing out that both types of enzymes can develop *de novo* and maintenance activities depending on genomic context (17). On the other hand, Ten-Eleven Translocation (TET) enzyme family members (TET1, TET2 and TET3) carry out demethylating activities by iteratively oxidizing 5-methylcytosine to 5-hydroxymethylcytosine, 5-formylcytosine and 5-carboxylcytosine, eventually reducing DNA methylation by base excision repair (21). Mechanistically, methylation physically prevents DNA binding proteins and transcription factor recruitment but, counterintuitively, promotes methyl-CpG-binding domain (MBD) proteins which cooperate with chromatin-remodeling-complexes to maintain euchromatic states (18, 21).

### 2.3 Histone post-translational modifications

Even though the most studied and characterized histone post-translational modifications (PTMs) are those involving small covalent modifications such as methylation of lysine residues, acetylation or arginine and phosphorylation, there is a plethora of other PTMs such as SUMOylation, ubiquitylation, ADP ribosylation, deamination and proline isomerization, among others (22, 23). The wide variety of identified modifications initially led to the elaboration of a histone code that could match each modification with a regulatory function in gene expression. However, additional research has revealed a higher level of complexity, where not a strict, but an intense, context-dependent crosstalk ultimately determines transcription activation of repression (23). Writer and eraser chromatin-modifying enzymes are in charge of depositing or withdrawing PMTs from histones, consequently altering chromatin structure. This highly dynamic process can take place by a direct effect of PTMs over intra- and inter-nucleosomal interaction as well as serving as docking sites for additional chromatin remodeling complexes that exert as the ultimate effectors of the histone PTM (18, 22, 24).

### 2.4 Non-coding RNAs and RNA modifications

Advances in human genome mapping have revealed than more than 80% of our whole genome has biological activity. However, it is estimated that only a 2% of transcribed loci are translated to proteins. Therefore, the vast majority of human transcriptome encodes (paradoxically) non-coding (nc)RNAs (25). Ranging from micro RNAs (miRNAs), small nucleolar RNAs (snoRNAs), small interferent RNAs (siRNAs), Piwi- interacting RNAs (piRNAs) to long non-coding RNAs (lncRNAs), ncRNAs are ribonucleic acid molecules expressed at lower ratios than protein-coding RNAs that exert essential silencing regulatory functions at both transcriptional and posttranscriptional level (18, 26). One of the best characterized ncRNAs are miRNAs: sequence-specific, single-stranded ≈ 22 nucleotide molecules that regulate gene expression by a decay in their target’s stability, hence resulting in protein downregulation (27). On the other hand, lncRNAs are defined as > 200 nucleotide sequences with implications in several processes such as cell proliferation, differentiation, migration and survival. Surprisingly, the functional mechanisms by which lncRNAs develop their regulatory functions are way more variated than other ncRNAs, and not enough studied (19, 28). Additional to their core functions, and similarly to what has been stated for DNA sequences, RNA molecules can also undergo several modification processes which alter their stability, transport or immune tolerance. More diverse when compared to DNA modifications, there are over 160 known RNA modifications equally controlled by Writer, Eraser and Reader enzymes. Among their most reported cases, it is noteworthy to highlight *N*^6^-methyladenosine (m^6^A), pseudouridine (Ψ), *N*^1^-methyladenosine (m^1^A), *N*^7^-methylguanosine (m^7^G), 5-methylcytidine (m^5^C) and 5-hydroxymethylcytidine (hm^5^C) (29).

## 3 Epigenetic immune reprogramming in hepatic antigen presenting cells in experimental models of chronic liver damage

Epigenetic alterations of HSCs in liver fibrosis evolution have been extensively studied elsewhere (30–32). However, the contribution of epigenetic changes to inflammatory immune cells functions and their effect in during liver fibrosis is an area of rising interest. Constituting the largest pool of resident tissue macrophages in human body, Kupffer cells develop a central role in both systemic and hepatic antigen response. Considering macrophage plasticity as one of their most relevant characteristics, a trait shared with epigenetic regulatory mechanisms, epigenetic KCs modulations has slowly generated particular interest. KCs epigenetic effectors involved in fibrosis and CLD progression are recapitulated in **Table 1**.

**Table 1:** Epigenetic effectors and affected pathways entailed in KCs function.

KCs						
Epigenetic effector	Target	Functional pathway	Targeting strategy	Targeting approach outcome	Disease model/sample source	Reference
DNA methylation						
DNMT3A DNMT3B	PSTPIP2 (5’-UTR)	IL-6, IL-1β and IL-10 expression. STAT1/6 phosphorylation	(AAV)–PSTPIP2 overexpression	Hepatic inflammation and fibrosis reduction	CCl4-induced mice hepatic macrophages	(33)
DNMT1	SOCS1 (Promoter)	JAK2/STAT3	5-azadC	Proinflammatory cytokine secretion prevention	RAW264.7 macrophages	(36)
DNMT1	Ptafr (Promoter)	PAF/PAF-R	5- azadC	Liver injury and inflammation amelioration, Th17-Treg rebalancing and Endothelial function improvement	Human hepatic non-parenchymal cells scRNA-Seq and CCl4-induced mice KCs	(34)
Histone modifications						
EZH2	H3K27me3 binding to TNF (Promoter)	NF-κB PKB/Akt	GSK126	Proinflammatory cytokine secretion prevention Acute liver failure hampering	Macrophages from liver failure patients and KCs from LPS+D-GalN injected mice	(41)
Set1 MLL	H3K4me3 binding to TNF and iNOS (Promoters)	Non-specified	SAMe MTA	Proinflammatory mediator downregulation	LPS treated mice and RAW264.7 macrophages	(42)
P300/CBP	H3K27ac H3K18ac	IL-6, TNFα, IL-1β, Ccl2, Ccl5 and Ccl3 expression	A-485	Inflammation and apoptosis reduction	LPS+D-GalN injected mice and RAW264.7 macrophages	(45)
KAT8	H4K16ac	IL-6, TNFα, Ccl2, TIMP1 and iNOS expression	n.s.	n.s.	Mx1-Cre; Mof f/f mice	(46)
ncRNAs						
miR-873-5p	GNMT	Caspase 3 activity. JNK phosphorylation	Anti-miR-873-5p	Liver function maintenance, fibrosis and inflammation amelioration	Cirrhotic/cholestatic patients and mice models	(95)
miR-142-5p (overexpression) miR-130a-3p (downregulation)	SOCS1	PPARγ	ASO-miR-142-5p LNA-miR-130a-3p	Macrophage profibrotic polarization inhibition	Macrophages and fibroblasts from healthy donors and CCl4-treated mice	(35)

5-azadC, 5-aza-2′-deoxycytidine; ASO, Antisense oligonucleotides; DNMT, Deoxyribonucleic acid-methyltransferase; AAV, Adeno-associated virus; LNA, Locked nucleic acid-modified oligonucleotides; miR, micro-ribonucleic acid; MTA, 5’-adenosilmetionine; n.s., not specified; ncRNAs, non-coding ribonucleic acids; PAF, Platelet Activating Factor; SAMe, S-adenosylmethionine.

While evaluating DNA methylation pattern and its influence in macrophage polarization, Yang et al. reported a hypermethylation of Proline-serine-threonine-interacting protein 2 (PSTPIP2) (shown by reduced representation bisulphite sequencing) in primary macrophages isolated from CCl_4_-treated mice. PSTPIP2 expression, mainly observed in KCs, was significantly reduced in CCl_4_-treated mice and LPS-stimulated RAW264.7 cell line. Downregulation was mechanistically associated to the hypermethylation of 5’-UTR regions (chr18:77843840-77843968) promoted by DNMT3A and DNMT3B. Liver-specific PSTPIP2 overexpression hampered hepatic inflammation and fibrosis by regulation of cytokine (IL-6, IL-1β, IL-10) expression and secretion, showing complex interactions with STAT1/6 phosphorylation (33).

Recent studies have also highlighted the interaction between resident liver macrophages and immune response, i.e., Platelet Activating Factor Receptor (PAF-R) expression has recently also been found to be epigenetically regulated in chronic liver disease progression, as reported by Methylation Array experiments carried out in FACS-sorted primary mouse macrophages isolated from CCl_4_ treated animals. *Ptafr* gene promoter demethylation has been associated to PAF-R overexpression observed in cirrhotic animals compared to controls, undergoing a consequent deterioration of vascular and liver function, assessed by extracellular matrix components deposition (i.e. collagen and actin), proinflammatory cytokine and chemokine expression and T cell subsets activation and proliferation. This correlation was mechanistically validated by *in vivo* experiments were DNMTs inhibition by 5-azadC injection in healthy mice mimicked cirrhotic phenotype and PAF-R overexpression (34).

As key regulators of several immune cell functions, ncRNAs play a crucial part in transcriptional macrophage regulation. *SOCS1* expression regulation is a remarkable example of the complex interplay of epigenetic mechanisms. Overexpression of miR-142-5p and downregulation of miR-130a-3p maintained a CCl_4_ profibrotic phenotype in macrophages which was recovered by miR-142-5p and miR-130a-3p inhibition and overexpression, respectively (35). Recently, a study reported that *SOCS1* promoter hypermethylation by DNMT1 was detected in RAW264.7 LPS-activated macrophages, and correlated to proinflammatory cytokine secretion downstream JAK2/STAT3 pathway (36).

Several studies have proposed the central participation of histone PTMs in HSCs activation during liver fibrosis and chronic liver disease progression, as extensively reviewed elsewhere (31, 37–39). Nonetheless, the role of macrophages’ chromatin structure modification by PTMs has not been extensively characterized yet; despite their key function in liver homeostasis and participation in the first steps of inflammatory response in liver damage and even fibrosis resolution (40). In this regard, H3K27me3 deposited by Enhancer of Zeste homolog 2 (EZH2) were identified as significantly upregulated in KCs from liver failure mice model, triggering the secretion of proinflammatory cytokines. A deeper epigenomic study identified a reduction of H3K27me3 enrichment in TNF promoter, thus increasing NF-κB and protein kinase B/Akt pathway activation upon LPS stimulation. In this context, EZH2 inhibition by GSK126 hampered acute liver failure in mice (41). Epigenetic effects of LPS-mediated macrophage stimulation have also been reported by activating histone marks associated to TNF promoter. H3K4me3 was found to be enriched in both TNF and Inducible Nitric Oxide Synthase (iNOS) promoters of KCs cells after LPS stimulation. In addition, H3K4 trimethyltransferases Set1 and MLL were also recruited to studied promoters upon stimulation (42).

Rather than histone tails methylation, histone acetylation has been described as a fundamentally activating posttranslational modification controlled by Histone Acetyltransferases (HATs) and Histone Deacetylases (HDACs), which respectively add or remove acetyl groups to histones. This is due to the fact that histone acetylation neutralizes the positive charge of lysine residues present in the nuclear proteins, reducing histone-DNA electrostatic interaction and facilitating a relaxed chromatin state (43, 44). The histone acetyltransferase p300 was identified as a key regulatory element in proinflammatory cytokine and chemokine expression (IL-6, TNFα, IL-1β, Ccl2, Ccl5, Ccl3) in an *in vitro* activated macrophage model (RAW264.7 cells stimulated with LPS), mechanistically explained by p300-catalysed H3K27 and H3K18 acetylation. Use of a specific p300/CBP inhibitor (A-485) suppressed macrophage inflammatory response in cell culture as well as in an *in vivo* mice model of liver injury (LPS + GalN intraperitoneal injection). In this context, KEGG pathways analysis resulting from whole liver tissue RNA-seq analysis reported an enrichment of inflammation and apoptosis-related pathways in diseased mice, which was suppressed by p300 HATs inhibition (45).

Within HATs superfamily of proteins, Lysine Acetyltransferase 8 (KAT8/MOF) has emerged as a notable component in several liver disease-associated phenomena. KAT8 non-redundantly acetylates H4K16ac, which is a prerequisite for additional H4 acetylation; and its deletion led to liver fibrosis in Mx1-Cre;Mof^f/f^ mice. In this study it was demonstrated that KAT8 regulated gene pathways in mice that are commonly dysregulated in human liver disease, highlighting an upregulation in gene sets involved in negative regulation of cell proliferation, response to LPS and response to cytokines. Interestingly, hepatocyte specific deletion of KAT8 had no apparent effects on liver physiology. However, simultaneous KAT8 deletion in both hepatocytes and Bone Marrow Derived Macrophages (BMDMs, a common *in vitro* model used for KCs study) occasioned an increase in hepatocytes apoptosis, altogether with an augmented expression of chemokines (CCL2), proinflammatory cytokines (IL-6, TNFα), profibrogenic (TIMP) and M1 macrophage markers (iNOS) by BMDMs (46).

Their participation in leukocyte recruitment and substance transport across porto-sinusoidal niche, in addition of being involved in early liver fibrogenesis, makes LSECs a relevant study target (see **Table 2**. for described epigenetic LSECs regulation). Regarding sinusoidal-cells epigenetic regulation, it is noteworthy to mention p300 HAT and its interacting proteins NF-κB and BRD4, which altogether increase C-C motif Chemokine Ligand 2 (CCL2) expression in primary mouse (control and CCl_4_-treated) LSECs. H3K27ac writing at *CCL2* promoter by p300 upregulated chemokine’s expression and led to CCR2^+^ monocyte/macrophage accumulation (studied both *in vitro* and *in vivo*), portal hypertension and liver fibrosis; pointing out p300 inhibition as a potential therapy for liver disease (47), highlighting the importance of histone acetylation in liver inflammation.

**Table 2:** Epigenetic effectors and affected pathways entailed in LSECs function.

LSECs						
Epigenetic effector	Target	Functional pathway	Targeting strategy	Targeting approach outcome	Disease model/sample source	Reference
Histone modifications						
p300-HAT	H3K27ac	NF-κB BRD4 Ccl2 expression	*p300*^LSECΔ/Δ^ mice	Fibrosis and portal hypertension amelioration CCR2^+^ macrophages accumulation reduction	Human LSECs and CCl4-treated mice	(47)
n.s.	H3K18ac	Fcgr2b Lyve1	n.s.	n.s.	Sodium arseniate-treated rats	(48)
SIRT1	p53ac	NOX2-dependent oxidative stress Progerin-associated premature senescence	AAV-SIRT1-overexpression	Inhibition of premature senescence and capillarization alleviation in LSECs	Primary LSECs from CCl4-treated rats	(49)
n.s.	H3K27ac	NF-κB Cxcl1, Cxcl2, Cxcl6 and Cxcl8 expression	3PO tamoxifen-inducible EC-specific *Hk2* knockdown mice (*Hk2*^fl/fl^/*Cdh5*^cre^-ERT2)	Neutrophil infiltration attenuation and portal hypertension reduction	Primary human and murine LSECs	(50)
n.s.	H3K27ac H3K4me1	Col4a1 Col4a2	n.s.	n.s.	Healthy and cirrhotic patient livers (RNA-Seq), human and murine LSECs and CCL4-treated mice	(51)
MLL1/BRG1	H3K4me3	Sp1/MLL1/BRG1/CAV1. eNOS-dependent NO production	siBRG1, siCAV1, siMLL EC-specific BRG1 deletion in mice (*Smarca4*^f/f^/*Cdh5*-Cre	Fibrosis attenuation eNOS activity and NO production recovery	TAA-treated mice and human immortalized LSECs	(52)

3PO (3-(3-Pyridinyl)-1-(4-pyridinyl)-2-propen-1-one); AAV, Adeno-associated virus; EC, Endothelial cell; n.s., not specified; si, Small-interferent.

Moreover, histone acetylation has emerged as a relevant factor involved in LSECs cell identity maintenance during CLD, according to recent publications. With this regard, liver fibrosis by chronic arsenic exposure has been associated to inhibition of H3K18ac and altogether linked to HSCs activation mediated by LSECs dedifferentiation. An *in vivo* experimental model reported a negative correlation between increasing NaAsO_2_ doses and H3K18ac writing in whole liver tissue samples. It was further revealed that H3K18ac enrichment was reduced in Fcgr2b and Lyve1 promoters: two relevant genes for LSECs differentiation phenotype maintenance, therefore suppressing their gene expression, whereas other endothelial specific receptors such as Stabilin-1 and Stabilin-2 were not affected (48).

HDACs are the other main protein superfamily controlling histone acetylation dynamic equilibrium. Sirtuin 1 (SIRT1) is a NAD^+^ dependent acetyltransferase part of the Class III HDACs category, found both in nucleus and cytoplasm cell compartments. SIRT1 downregulation associated to LSECs defenestration due to CCl_4_ induced liver fibrosis was reported in an experimental model *in vivo*. In addition, *in vitro* experiments indicated that LSECs capillarization was mediated by p53 acetylation and aggravated by oxidative stress and progerin-associated premature cell senescence, mitigated by p53 deacetylation through SIRT1 overexpression, thus maintaining LSECs fenestra and functional phenotype (49).

TNF-α and its downstream transcription factor NF-κB have also been related to expression inducing histone PTMs and liver fibrosis complications; such are the cases of collagen type IV (COL4) and C-X-C motif chemokine Ligand 1 (CXCL1) expression in LSECs (50). COL4 (a major component of basement membrane associated with sinusoidal architecture remodeling and portal hypertension) was upregulated in LSECs from mouse cirrhotic liver samples. Putative *Col4a1* and *Col4a2* enhancer regions were identified through H3K27ac and H3K4me1 enrichment analysis by tagmentation-assisted fragmentation of ChIP-Seq in mouse livers (51). CXCL1 overexpression functional capacity was evaluated, registering a promotion in neutrophil infiltration, portal hypertension and liver fibrosis in a partial ligation of inferior vena cava mice model (50).

Some innovative studies have pointed to LSECs histone methylation modification as a process involved in endothelial function and CLD progression. Precisely, endothelial-specific deletion of the chromatin remodeling protein Brahma-Related Gene 1 (BRG1) ameliorated liver fibrosis progression in an *in vivo* mice experimental model generated by thioacetamide injection. Mechanistically, BRG1 interacted with MLL1 (an H3K4 trimethyltransferase), modulating H3K4me3 enrichment in Caveolin-1 (CAV1) promoter. Exhaustive analysis combining experiments in human immortalized LSECs and animal experiments revealed that Sp1 recruited BRG1 to the CAV1 promoter and activated its transcription in LPS challenged LSECs, ultimately modulating eNOS-dependent nitric oxide production and endothelial function (52).

Regarding DCs, bone marrow-derived Dendritic Cells (BMDCs) stimulated with DC growth factors (GM-CSF, Flt3L) have helped understand relevant epigenetic regulators in this cell population (53, 54). This is the case of some epigenetic readers such as Bromodomain and Extra-Terminal domain protein family (BET) and the bromodomain-harboring protein Speckled 140 KDa (SP140). Both BET and SP140 are associated to the “reading” process of acetylated lysine residues from histone tails, ultimately recruiting transcription factors and chromatin remodeling complexes that regulate gene transcription (55). Specifically, BET bromodomain inhibition by I-BET151 treatment reduced LPS-stimulated murine BMDCs maturation, as reported by a reduced inflammatory cytokine response (IL-6, IL-12A, IL-12p70, IL-10 and IFNγ) and decreased expression of immune-response associated receptors such as TLR-2/9. Functional capacity of DCs was also confirmed after assessing their ability to promote regulatory T cells and ultimately regulate antigen specific T cell proliferation. BET inhibition was found to be also involved in DCs maturation and mature-associated functions: I-BET151 treatment reported a reduction in the expression of DCs maturation markers CD80 and CD86; in addition, BET inhibition in mature BMDCs increased Foxp3 induction in naïve T cells, generating functionally suppressive CD25+ Foxp3+ T cells (55).

In addition, epigenetic “writers” have also been reported to play a pivotal role in DCs maturation, function and influence in liver failure outcome. This is the case of the increase expression of H3K27me3 and its writer EZH2 observed in a mouse model of Fulminant Hepatic Failure (FHF) failure by Propionibacterium Acnes intravenous injection, specially reported in liver CD11c^high^MHCII^high^ DCs. In this work, EZH2-DC specific deficiency (Ezh2^D-/-^ mice) ameliorated ALT and AST levels in mice serum, nodules and granuloma formation and severe lymphocyte infiltration and CD4+ T -cell response (CXCR3, CCR7, CD44, CD62L downregulation), indicating an overall reduction in liver injury, but also in mortality after LPS challenging. In addition, a reduction in CD80, CD86 and MHCII expression in CD11c^+^ DCs reported an increased dendritic cell maturation. Pharmacological inhibition of EZH2 with specific molecule GSK126 reproduced the aforementioned results regarding amelioration of bacteria-induced liver injury (56).

Finally, post-transcriptional modifications of mRNA and their effects in mRNA stability, translation or premature degradation, are involved in epigenetic regulation of multiple DCs processes. Particularly, mRNA N6-methyladenosine (m^6^A) modification has arisen as a relevant regulator of DCs activation and function. RNA methyltransferase Mettl3-mediated mRNA m^6^A methylation has been shown to *in vitro* (BMDCs) and *in vivo* promote DC maturation (CD40, CD80 and IL-12 expression) and DC-related T-cell response (TLR4/NF-κB pathway, IL-6 and IL-12b expression) involving m^6^A reader protein Ythdf1 (57, 58). mRNA m^6^A methylation is also a great example of the complex interplay between different epigenetic regulatory mechanisms, as it also affects other epigenetic effectors stability such as ncRNAs. Specifically, lncRNA lnc-Dpf3 degradation is prevented in BMDCs by m^6^A demethylation as part of a pathway upstream initiated with CCR7 stimulation, ultimately suppressing DC migration via HIF1α-dependent glycolysis inhibition. Further *In vivo* experiments in lcn-Dpf3-Deficient mice confirmed this experiments, additionally showing expanded Th1 and Th17 cell compartments (59). Epigenetic DCs regulatory players cited in this section are compiled in **Table 3**.

**Table 3:** Epigenetic effectors and affected pathways entailed in DCs function.

DCs						
Epigenetic effector	Target	Functional pathway	Targeting strategy	Targeting approach outcome	Disease model/sample source	Reference
Histone modifications						
BET	H3K18ac	IL-6, IL-12a, IL-12p70, IL-10, IFN-γ, FoxP3 TLR2 and TLR9 expression	I-BET151	Reduction of DCs maturation and cytokine response. CD25+FoxP3+ cells proliferation	Human and mouse derived dendritic cells	(55)
SP140	n.s.	TNF, IL-1β, IL-6, IL-10, IL-8 production. GM-CSF, CCL6, CCL5, CCL9, CXCL10, CCL1 expression	GSK761, siSP140	DCs tolerogenic properties enhancement	Human dendritic cells *in vitro* generated from CD14+ isolated monocytes	(102)
EZH2	H3K27me3	ALT and AST production TNF, IFN-γ, IL-6 production CD4+ T-cell response RUNX1 expression	GSK126, DC-specific deficient Ezh2^-/-^ mice	Liver injury severity amelioration, reduced mortality and T-cell response DCs proinflammatory functions and maturation inhibition	*Propionibacterium acnes* and LPS-treated mice	(56)
ncRNAs						
Lnc-Dpf3	HIF1α	CCR7/m^6^A	DC-specific lnc-Dpf3^-/-^ mice, siDpf3, Dpf3^OE^	Increment of severe inflammation Th1 and Th17 proliferation	Primary bone marrow-derived DCs and contact hypersensitivity mice model	(59)
RNA modifications						
Mettl3	m^6^A	Ythdf1 expression CD40, CD80 and IL-12 expression TLR4/NF-κB induced cytokine production	Ythdf1^-/-^ mice Specific exon-deficient Mettl3 mice	Impaired phenotypic and functional DC maturation Reduced T-cell response stimulating capacity	*Mettl3*KO mice, *Lysteria monocytogenes* infected mice and primary bone marrow-derived DCs	(57)

5-azadC, 5-aza-2′-deoxycytidine; DCs, Dendritic Cells; m^6^A, N6-methyladenosine; n.s., not specified.

## 4 Evidence on human liver disease and clinical relevance

Alterations in epigenetic mechanisms controlling gene expression are now widely recognized as key contributors to liver disease progression, arising the possibility of their use as potential therapeutical targets. Epigenetic influence in cancer development is a well-known example, with several studies addressing the therapeutical implications of druggable epigenetic targets intervention (60–70).

On the other hand, alcohol related liver disease is one of the most prevalent forms of liver disease in the world, encompassing a wide variety of etiologies such as alcohol-related fatty liver, alcohol-related hepatitis/steatohepatitis, alcohol-related cirrhosis and hepatocellular carcinoma. Given the limited treatment options, epigenetic targeting gives an interesting therapeutical option with clinical possibilities (71–76). A similar situation involving characterization of epigenetic and epitranscriptomic players has developed in study of non-alcoholic fatty liver disease (77–79), inflammatory liver diseases (80), metabolic dysfunction associated steatotic liver disease (81–85) and viral hepatitis (86–88).

Liver toxicity of epigenetic drugs has been an important field of study. Reports of clinically overt hepatotoxicity with some of these agents have been documented (89–94). It is possible that adverse effects may be found during epigenetic drugs trials due to their pleiotropic effects on gene regulation, their non-specific activity on undesired proteins and the lack of cell-specific targeting that emerges with systemic administration, being particularly relevant when thinking about patients with impaired liver function.

**Figure 3:**
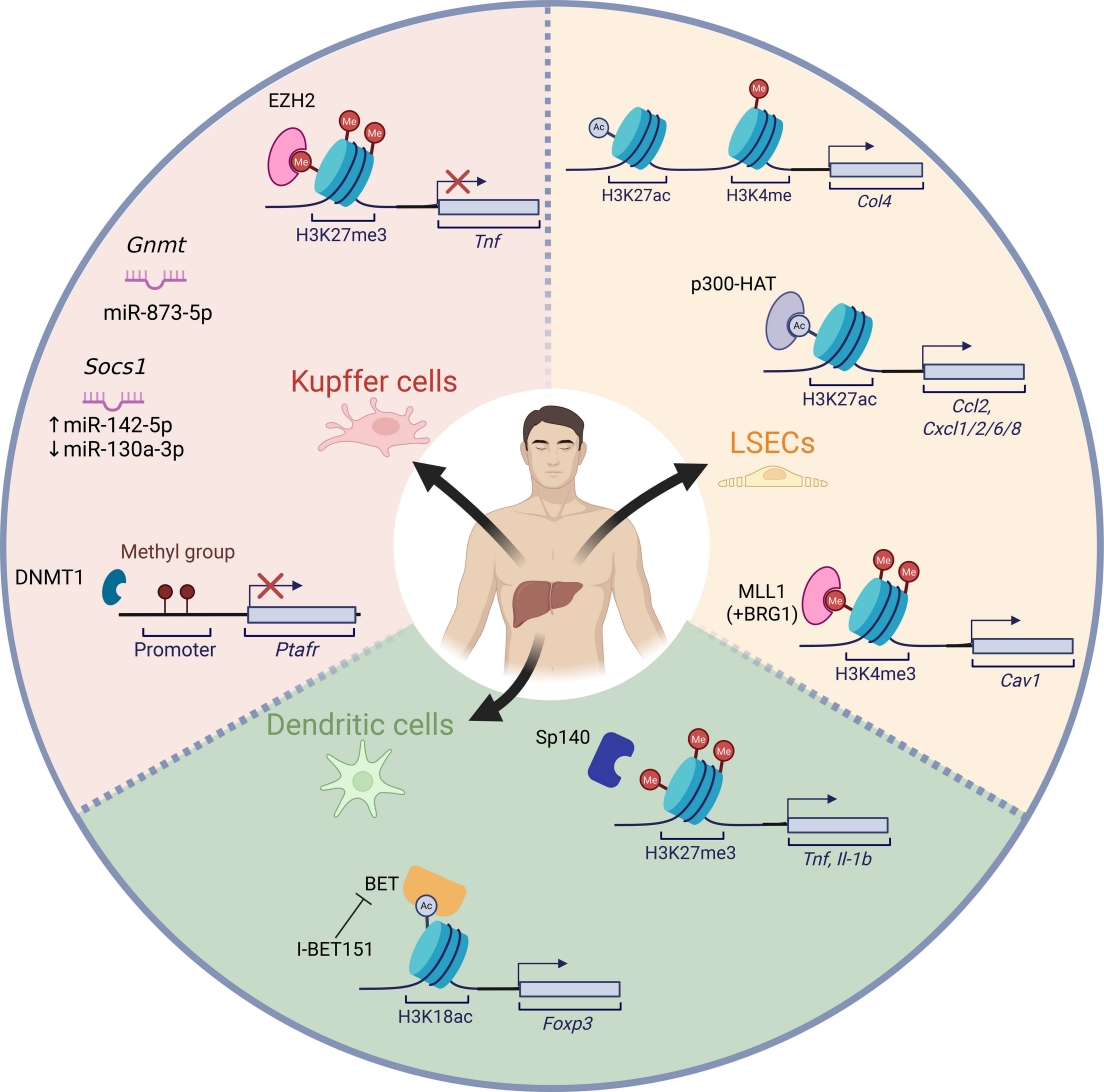
Epigenetic mechanisms with evidence reported in human hAPCs. Epigenetic gene expression regulatory mechanisms gathered either in patient samples, primary cells or immortalised cell lines with in vivo or in vitro evidence.

Nevertheless, the specific effect of epigenetic modifiers on hepatic antigen presenting cells reviewed herein during advanced chronic liver disease remains to be elucidated (**Figure 3**). As shown in murine KCs, H3K27me3 bound by EZH2 was significantly upregulated in Peripheral Blood Mononuclear Cells (PBMCs) isolated from Acute on Chronic Liver Failure (ACLF) patients (41). In human KCs, Glycine N-methyltransferase (GNMT) is the most abundant liver methyltransferase and mediates transmethylation flux by competition with conventional DNMTs. A potential downregulation of GNMT by miR-873-5p was associated with loss of liver function and fibrosis progression in cirrhotic and cholestatic patients as well as several cholestasis and fibrosis mouse models. Anti-miR-873-5p therapy in bile duct ligation and *Mdr2^-/-^* mice recovered GNMT expression and ameliorated inflammation and fibrosis (95). Also, the overexpression of miR-142-5p and downregulation of miR-130a-3p was observed in CD68^+^ macrophages from cirrhotic patients. Both miRNAs were identified as regulators of M2 polarization through SOCS1 and PPARγ. In addition, mir-130a-3p downregulation was correlated to IL-4 induced H4 deacetylation in human macrophages (35).

When evaluating LSECs, arsenic poisoning-liver damage epigenetic mechanisms have also been reported in humans, where liver oxidative stress caused by chronic arsenic exposure reported an up-regulation of miR-155 by hampering the blocking effect of AUF1 on Dicer1. SOD1 3’-UTR region was ultimately targeted by miR-155, described as a relevant T cell infiltration regulator during chronic, low-grade inflammation involved in different liver diseases (96–99). Additionally, increased H3K27ac and H3K4me1 at both enhancer and promoter regions of *Col4a1* and *Col4a2* analyzed in human cirrhotic livers, indicated a potential novel epigenetic regulatory mechanism of COL4 in LSECs (51). Moreover, H3K27ac enrichment analysis by chromatin immunoprecipitation reported an overrepresentation of studied activating PTM in *CXCL1* promoter region of primary human LSECs under mechanical stress conditions. In this study, the deposition of H3K27ac in several CXCL family genes (i.e., *CXCL1/2/6/8*) and additional experimental evidence (Circularized, Chromosome Conformation Capture, 4C and ChIP-Seq) hinted toward a spatial reorganization of nuclear chromatin, enabling H3K27ac recruitment to a super-enhancer region, resulting in NF-κB binding to *CXCL1* promoter (50).

Hepatic dendritic cells are unquestionable key players in hepatic immune homeostasis and liver disease progression due to their role as professional antigen presenting cells. However, owing to the limited number of this specific cell type that can be successfully isolated from the liver, the functional characterization of such an important immune cell population has not been fully completed yet. Nonetheless, epigenetic regulators of DCs’ cell activity and maturation have been studied in non-hepatic DCs obtained from monocytes (mdDCs) stimulated with DC growth factors (100, 101). As also studied in mice, I-BET151 treatment reduced proinflammatory cytokines, TLRs and co-stimulatory molecules expression as maturation markers in LPS-challenged mdDCs (55).

Also, LPS challenging of human mdDCs (*in vitro* generated DCs by CD14+ monocyte isolation and GM-CSF + IL-4 stimulation) led to an SP140 increased expression and recruitment to transcription start sites of pro-inflammatory cytokines, highlighting IL-1βb and TNF. This relation was validated by a reduction in proinflammatory cytokine and chemokine expression (TNF, IL-6, IL-1β, IL-8, GM-CSF, CCL3, CCL5, CCL9, CXCL10 and CCL1) after GSK761 or SP140siRNA DCs treatment. mdDCs maturation was also inhibited by GSK761, evaluated as a reduction in the expression of surface maturation markers, co-stimulatory molecules and antigen presenting molecules including CD83, CD80, CD86, CD1b. Effect of GSK761 in DCs functional T-cell stimulating capacity was studied, showing reduced TBX21 and RORA expression and increased FOXP3 expression, indicating a polarization toward regulatory T-cell subsets, therefore potentially enhancing the tolerogenic properties of DCs (102).

Due to the current scarcity of studies specifically dissecting the epigenetic regulation of hepatic antigen-presenting cells (APCs) across chronic liver disease progression, there are relatively few datasets that combine APC-focused analyses with cutting-edge multi-omic technologies such as single-cell RNA sequencing (scRNA-seq), spatial transcriptomics, or ATAC-seq in this precise context. Most state-of-the-art work instead applies these platforms more broadly to liver fibrosis, NASH/MASH, HCC, cirrhosis or mixed non-parenchymal cell (NPC) compartments, often without resolving APC epigenetic programs in detail. For example, scRNA-seq studies in human NASH and NASH with fibrosis have mapped macrophage and other NPC subpopulations and their transcriptional signatures at single-cell resolution but focus mainly on transcriptional rather than epigenetic regulation (103–107). Spatial transcriptomics and multi-modal single-nucleus approaches integrating RNA and chromatin accessibility (snRNA-seq + snATAC-seq) have begun to chart gene regulatory networks and spatial niches driving human cirrhosis and liver fibrosis (108–111), yet chromatin accessibility data in liver disease remain relatively sparse and are not APC-centric (111, 112). More generally, multiomic approaches combining scRNA-seq with single-cell ATAC-seq are highlighted as powerful tools to link transcriptional states to epigenetic regulation in NASH and other liver diseases, but their application has so far concentrated on hepatocytes, stellate cells and broad NPC compartments rather than on dedicated hepatic APC subsets (11, 111, 113–116). This gap contrasts with the rapidly expanding epigenetics literature in chronic liver disease (including NASH, viral hepatitis, fibrosis and cirrhosis) at the bulk level (28, 117–120).

## 5 Future perspectives

As evidenced in several other liver diseases such as fibrosis and cancer, emerging experimental evidence indicate a relevant role for epigenetic regulation of hepatic antigen presenting cells in chronic liver disease. Experimental *in vitro* and *in vivo* results reviewed herein show the potential benefit of targeting epigenetic mechanisms to ameliorate KCs activation, LSECs proinflammatory secretome polarization, DCs maturation and mature-associated functions during chronic liver disease. Nevertheless, given the complexity of underlying epigenetic mechanisms involved in those processes and the intense crosstalk between different epigenetic events in gene regulation, further studied are required to achieve a clustering consensus. Potential adverse effects may be found during epigenetic drugs trials due to their pleiotropic effects on gene regulation, their non-specific activity on undesired proteins and the lack of cell-specific targeting that emerges with systemic administration (being particularly relevant when thinking about patients with impaired liver function).

Importantly, although a key conceptual advantage of epigenetic regulation is its potential reversibility, the extent to which disease-associated epigenetic programs in hAPCs remain functionally reversible in advanced stages of chronic liver disease remains uncertain. In immune cells, persistent inflammatory and metabolic cues can establish relatively stable epigenetic programs that may not be readily reset by short-term pharmacological inhibition of individual epigenetic effectors targeting. In this context, pharmacological modulation of single epigenetic regulators may only partially reset transcriptional programs that drive pathogenic phenotype of hAPCs; or may generate intermediate phenotypes rather than complete restoration of homeostatic immune tolerance. Moreover, broad epigenetic modulation may unintentionally disrupt homeostatic programs required for normal cell function in the liver, potentially aggravating immune-mediated injury. These considerations highlight that the theoretical reversibility of epigenetic marks does not necessarily translate into predictable or fully controllable therapeutic outcomes *in vivo*.

These potential pitfalls, and the limited knowledge on epigenetic regulation of hAPCs underscores the need for future studies that directly profile hAPC specific chromatin landscapes using single cell and spatial epigenomic technologies (e.g. sc/snATAC-seq, multiome, spatial transcriptomics) along the spectrum of chronic liver disease. Such approaches will be essential to determine whether disease-associated epigenetic states in KCs, LSECs and DCs represent transient regulatory programs or more stable chromatin configurations, and to identify context-dependent epigenetic targets that could be modulated with greater precision and safety.
